# Assessing the Impact of a Culturally Congruent Perinatal Home-Visiting Program on Gestational Age at Delivery for Black Women

**DOI:** 10.1089/heq.2024.0076

**Published:** 2024-09-12

**Authors:** Erin Snowden, Deborah F. Perry, Rabiyah Amina, Bryan Shaw, Aza Nedhari

**Affiliations:** ^1^ Data and Social Impact, Mamatoto Village, Inc, Washington, District of Columbia, USA.; ^2^ Center for Child and Human Development, Georgetown University, Washington, District of Columbia, USA.; ^3^ Center for Global Health Practice and Impact, Georgetown University, Washington, District of Columbia, USA.; ^4^ Executive Director and Co-Founder, Mamatoto Village, Inc, Washington, District of Columbia, USA.

**Keywords:** perinatal health disparities, culturally congruent care, community-based interventions

## Abstract

**Purpose:**

There is a Black maternal health crisis in America, with significant racial disparities in birth outcomes for Black women in Washington, DC. Programs designed to reduce these inequities must intentionally address the role of systemic racism and the ongoing legacy of oppression that is endemic to traditional perinatal care services. This article describes the findings from the quantitative analysis of an innovative perinatal program (Mothers Rising) designed by and for Black women in the Washington, DC, metropolitan area that was part of a larger mixed methods study.

**Methods:**

Using data provided by a Medicaid managed care organization that insured program participants and women who did not receive Mothers Rising, program participants (*n* = 102) were matched with a group of Black women who did not receive the program (*n* = 102) using propensity scores matching. Perinatal outcomes were extracted from electronic health records from the managed care dataset, including birth weight and gestational age.

**Results:**

Statistical analyses of the differences in birth outcomes between program participants and their matched peers demonstrated small but statistically significant differences in gestational age, favoring the Mothers Rising group.

**Conclusions:**

This study adds to the evidence base for the effectiveness of culturally tailored interventions to successfully address persistent racial disparities in Black women’s perinatal health outcomes that result from persistent racism. Hyperlocal, community-developed home-visiting programs, such as Mothers Rising, should be funded to sustain impact and optimize maternal health outcomes.

## Introduction

There remain persistent disparities in infant mortality between Black and White babies, primarily driven by differences in rates of prematurity and preterm delivery. In 2021, the overall rate of preterm birth was 10.5%. Still, for non-Hispanic Black women, it was 14.8%.^1^ These racial differences are largely independent of education and socioeconomic status: Black, well-educated women who receive high-quality prenatal care starting in the first trimester have worse outcomes than similarly educated White peers.^2^ To close the gap, more must be done to identify the root causes of preterm delivery and develop interventions that can support Black women during the perinatal period.

Although researchers have been working on this question for decades, pinpointing modifiable causes of prematurity has been challenging.^3^ The prevailing view is that preterm delivery is initiated by a confluence of biological and epigenetic factors.^2^ Recently, there have been several notable shifts in the empirical literature: a call to shift the focus from race to racism and a focus on stress as an etiologic variable needing to be unpacked.^4,5^

Because of systemic racism, Black women are exposed to higher levels of stress across their lifespans. Discrimination, microaggressions, and other downstream environmental and psychosocial exposures that result directly from racism in America may, in part, explain the disparities in preterm delivery for Black women across the socioeconomic spectrum.^3^ Acute and chronic stressors are thought to impact multiple physiological functions (e.g., heart rate, endocrine, and immune functions), which could then cascade to influence uterine and placental functioning.^3^ It is well established that living with racism has physiological consequences for Black people, and the effects of racism on Black women’s bodies have been described in the literature with constructs such as “stress age,” “weathering,” and “allostatic load.”^3,6,7^

Obstetric racism—as a contextually specific manifestation of White supremacy and sexism—has a particularly long history of harming Black bodies.^5^ The field of obstetrics and gynecology built its disciplinary knowledge base through harmful and exploitative procedures on enslaved Black people. Further eroding safe places for Black women and birthing people, White male physicians and White female certified nurse midwives undermined the practices of Black “Grand Midwives.”^8,9^ These converging trends collide in what Scott and Davis refer to as “the afterlife of slavery”^5^, p 682, which still casts a shadow on Black birthing people’s experiences in the current perinatal care systems in the United States. Implicit bias, denial/dismissal of patients’ levels of pain, and other patterns of health care provider communication and behaviors create an environment where early signals are often missed.

Within this environment, a Black woman-led organization was formed, and a culturally congruent initiative was created to recruit and train the next generation of birthworkers and maternal health care providers to serve the women and birthing people of the Washington, DC, Region. The Black communities that have existed for years east of the Anacostia River—only a few miles from the U.S. Capitol—have some of the highest rates of maternal and infant mortality in the District of Columbia.^10^ These rates are driven in large part by differences in preterm delivery: rates of preterm birth for Black women in Washington, DC, are 1.5–2 times higher than for White women.^11^ In 2022, the national rates, where Black women experience preterm birth 14.6% of the time, almost 50% more likely than White women, are not dissimilar to racial disparity observed in Washington, DC, and 1.4 times higher than Hispanic women.^12^

The Mothers Rising Home Visiting (MRHV) program was created to address these disparities and deploy a workforce equipped to disrupt the caustic experiences of their clients during their perinatal experiences. Community-based, culturally congruent, perinatal care, and comprehensive social initiatives do not offer an absolute solution to the effects of systemic racism in maternal and child health. However, MRHV is a remedy focused on the impacted community by offering protection from harmful practices and policies of the industries contributing to social determinants of health. MRHV offers a way to improve perinatal outcomes by shifting the Black community away from systems that have divested from ensuring they experience the best outcomes toward whole health and wellness.

This article begins with a description of the MRHV program that was created by and for Black women to interrupt the pernicious and persistent influences of structural racism on maternal and child health outcomes. We then share some quantitative findings from a matched comparison group of program participants who were part of a larger mixed methods evaluation. We conclude with a review of similar efforts underway in other communities seeking to close the racialized perinatal health disparities that exist across the United States.

### The intervention

Mamatoto Village (MV) is a Black-led, community-based perinatal health organization in the District of Columbia. Through a host of services, MV serves the community by providing the affirmation, knowledge, and tangible resources necessary to realize a healthy pregnancy, empowering birth, healing postpartum, and social mobilization for Black families. The MRHV program is the agency’s proprietary service delivery initiative that prioritizes providing perinatal support services to Medicaid-eligible District of Columbia residents, with a special focus on those who identify as Black or African American. The tenets of culture, familial and social connection, and community care are reflected in MRHVs theory of change, which highlights four key components: rigorous training, cultural reflection, social proximity, and a three-generation approach.

- Rigorous training. Women from the communities that the MRHV program serves—including former participants—are recruited to join a rigorous and immersive training cohort. With over 160+ h of didactic education, the most unique aspect of Mamatoto’s comprehensive workforce training is focused on the specialty of perinatal health infused in a community health worker model grounded in reproductive and birth justice. This training has offered almost 200 Black trainees a stepping stone into economically sustainable careers that encourage community transformation.
- Cultural reflection. The program design is grounded in a model of cultural humility and congruence. Perinatal community health workers (PCHWs) are highly trained community members residing in, or have close connections to, the prioritized community and therefore dedicated to delivering culturally specific accompaniment and respectful care. Although it is impossible to assume that the staff will reflect every aspect of culture represented among clientele since the Black experience is not a monolith, barriers to engagement are mitigated due to shared self-identification with Black culture. The shared language, arts, food, social norms, and cultural expression organic to MRHV staff, administration, and clients are reflected throughout every aspect of the MRHV program, from interpersonal communication to educational materials, creating an environment of familiarity, comfort, and trust.
- Social proximity. The MV staff comprise people who mirror the experiences, family dynamics, and neighborhoods of those they serve. They strive to make the transition to parenthood self-determined and rooted in nurture, nonjudgmental support, advocacy, and evidence-based information, realizing their work impacts and uplifts the entire community. PCHWs are committed to meeting the needs of their clients where they are and providing services in various settings, including the home, providers’ office, general community, or MV offices.
- Three-generation approach. The mother–baby dyad does not exist in a silo. The recognition of the intergenerational transmission of health, health literacy, well-being, wealth, and social status is reflected in the three-generation approach, which is an upstream solution requiring holistic interventions to optimize parent and child bonding. It emphasizes the need to respectfully engage, educate, and empower the woman and the familial support system, typically grandparents or other close elders, to facilitate a move for the entire family toward self-determination, wellness, and joy.

Before entering the field, PCHWs are fully equipped to provide high-quality care through a holistic lens, understanding that the outcomes of the perinatal period are affected by factors that expand beyond the physiological aspects of pregnancy and childbirth. The successful completion of the training provides the foundational knowledge necessary to implement a scope of practice reminiscent of the Grand Midwives that expands well beyond the scope of present-day doulas, equally prioritizing pregnancy and health-related support alongside the recognition and intervention on the psychosocial components of the social determinants of health on a timeline that spans the prenatal period to a minimum of 12 weeks postpartum.

The MRHV journey begins with the client taking an in-depth psychosocial assessment that is used to cocreate an individualized care plan that assists the client and PCHW in identifying the prioritized areas of need to develop realistic goals and a pathway to their realization. For an average 30-week enrollment, PCHWs work in teams to provide wraparound care to each client with education and support related to their pregnancy, identified social needs, and health and wellness concerns, including fitness, stress management, mental health interventions, and lactation support. Certain services, including breastfeeding anticipatory guidance, postpartum planning, 24-h postpartum care, and discharge planning, are offered to each client on a standardized timeline. The cadence of these encounters is purposefully designed to ensure the family receives direct care at milestones in the perinatal period where critical decision-making, mental health vulnerabilities, and advocacy needs are elevated. Clients are also provided tangible resources such as personal hygiene and baby care items, emergency food supplies, and safe sleep items. Furthermore, clients can build a meaningful and supportive community with other enrollees during group sessions. By design, clients who receive services through MRHV are often inspired to enroll in the PCHW training and join MV to provide these necessary services within the community.

## Methods

MV, as an organization, has made a strong commitment to data-driven service delivery. Building upon their own research and evaluation efforts, a partnership with the Georgetown University Center for Child and Human Development was initiated with grant funds provided by a competitive award from the DC Department of Health. A concurrent mixed methods evaluation was designed with the university partners that centered on participants’ lived experiences and added a rigorous comparison group for the MRHV program participants. The study design included in-depth, semi-structured interviews with a group of program participants who had a prior birth without support from the MRHV program; in this way, each participant had lived experiences within and without the program. The findings from these qualitative analyses will be available in a separate report.

For the quantitative analyses reported in this article, the research partnership also built upon a long-standing relationship that MV had with one of the Medicaid Managed Care Organizations (MCO) that serves families in the District of Columbia. The Medicaid MCO agreed to share deidentified claims data on a large sample of birthing people who received coverage for their pregnancies during the study period, including women who received services from MRHV. A university-based statistical consultant unfamiliar with the MRHV used these data to create a comparison group for the program participants using propensity score matching, as described below.

### Quantitative data collection and analysis

Propensity score matching was used to create a comparison group of pregnant people similar to the Mothers Rising participants. The dataset from our Medicaid MCO partner contained 3,703 birthing people who delivered between January 1, 2019, and December 31, 2020, in District of Columbia area hospitals. These data included specific diagnostic and procedure listings (International Classification of Diseases, 10th revision, codes) for the 270 days before delivery and 90 days postpartum, reflecting the typical time frame clients are enrolled in MRHV; delivery outcomes, including level of care, birth weight, gestational age at delivery, and delivery mode; and social determinants of health captured in providers’ visit notes.

#### Variables included in the quantitative analysis

Four covariates that have been associated with differences in perinatal health outcomes were selected to be used to create the matched comparison group for the MRHV participants: maternal age at delivery, race, delivery hospital (a proxy for health care systems/policy), and zip code of residence (as a proxy for neighborhood-level risk factors).^13^ Maternal delivery age was categorized into five-year categories (14–19, 20–24, etc.). Maternal race was categorized according to U.S. census groupings. Delivery hospitals were clustered by geography (DC-based, Maryland, and Virginia hospitals). Zip codes were categorized into four health outcome risk pools based on the SocioNeeds Index (recently renamed the Healthy Equity Index) by calculating quartiles from the index values that ranged from 0 to 100.^14^ The Healthy Equity Index, formerly the SocioNeeds Index, is an analytic tool that encapsulates health and socioeconomic indicators into a composite score to identify possible areas for action. In the current analysis, respondent participation in the MRHV program (coded yes/no) was the primary predictor.

All outcome variables were derived from MCO claims data and reflect the most common outcomes of interest to policymakers, payers, and other stakeholders. Birth weight and gestational age were captured as continuous outcomes, and low birth weight (<2,500 g), preterm birth (<37 weeks), and level of care were analyzed as dichotomous outcomes. Gestational age is a continuous variable and signifies the estimated time (in weeks) between conception and birth. This variable was also dichotomized to assess preterm births (<37 weeks) versus complete-term births (<37 weeks). Birth weight is a continuous variable and is a baby’s weight (in grams) at birth. The variable was also dichotomized to assess low birth weight (<2,500 g) versus normal birth weight (<2,500 g). A final outcome considers whether a baby was enrolled in the neonatal intensive care unit (NICU) or the Well Baby Nursery after birth. It is a dichotomous variable (coded no = NICU, yes = Well Baby Nursery).

#### Quantitative analysis

A 1:1 propensity score matching process created a comparison group for the MRHV participants using the covariates listed above. Specifically, regression with the teffects psmatch function of STATA 17^®^ (caliper 0.03, no replacement) was performed using the following parameters: maternal delivery age, race, delivery hospital, and zip code risk category. The teffects psmatch function provides the average treatment effect on the treated. To increase the robustness of the propensity score method, unadjusted and adjusted linear (continuous dependent outcomes) and logistic (dichotomous dependent outcomes) were performed, with adjustments for maternal delivery age, race, delivery hospital, and zip code risk category. All analyses were conducted using Stata 11 (Stata Corp, College Station, TX).

Statistical differences comparing respondent characteristics based on MV program participation were examined through chi-square tests for categorical variables and *t*-tests for continuous variables for the full sample (MV, *n* = 102; non-MV, *n* = 3,601). Propensity score matching methods were used to investigate the effect of MV program participation on key programmatic outcomes: birth weight and gestational age—continuous outcomes—and low birth weight (<2,500 g), preterm birth (<37 weeks), and level of care (NICU vs. Well Baby Nursery)—dichotomous outcomes.

Table 1 demonstrates that the use of propensity scores resulted in a well-matched comparison group for the MRHV participants. The mean (standard deviation) age at delivery among the MRHV sample was 27.7 (5.7) years, compared with 28.1 (5.5) years for the matched controls. The majority of both the MRHV and matched control sample were Black (81.9%), most (58.8%) lived in high-risk [BS1] zip codes in Washington, DC, and large majorities delivered at the Washington Hospital Center (35.3% of the MRHV sample; 36.3% of the matched control sample) or other Washington, DC, hospitals (61.8% of the MRHV sample; 60.8% of the matched control sample). There were no statistically significant differences comparing sociodemographic characteristics between the MRHV and the matched control sample.

**Table 1.** Variables Used to Create the Propensity Scores Matched Sample for the Mothers Rising Home Visiting Program

Characteristics	MRHV program participants (*n* = 102)	Matched control (*n* = 102)	*p*-value^a^	Unselected control pool^b^ (*n* = 3,499)
Covariates				
Maternal age at delivery (in years)			0.643	
Mean (SD)	27.7 (5.4)	28.1 (5.5)		27.7 (5.7)
Maternal age categories			1.000	
	Number (Percent)	Number (Percent)		Number (Percent)
14–19	3 (2.9)	3 (2.9)		247 (7.1)
20–24	24 (23.5)	25 (24.5)		809 (23.1)
25–29	41 (40.2)	41 (40.2)		1,214 (34.7)
30–34	21 (20.6)	20 (19.6)		775 (22.2)
35–39	11 (10.8)	11 (10.8)		267 (10.5)
40–51	2 (2.0)	2 (2.0)		87 (2.5)
Race			0.920	
Black	93 (91.2)	93 (91.2)		2,848 (81.4)
Hispanic	0 (0.0)	0 (0.0)		61 (1.7)
White	1 (1.0)	1 (1.0)		45 (1.3)
Asian/Pacific	0 (0.0)	1 (1.0)		15 (0.4)
American Indian	0 (0.0)	0 (0.0)		10 (0.3)
Middle Eastern/North African	1 (1.0)	0 (0.0)		6 (0.2)
Unknown	3 (2.9)	3 (2.9)		318 (9.1)
Other	1 (1.0)	1 (1.0)		130 (3.7)
Declined to state	3 (2.9)	3 (2.9)		66 (1.9)
Residence (zip code risk level)			1.000	
Lowest risk	3 (2.9)	3 (2.9)		91 (2.6)
Medium low risk	22 (21.6)	22 (21.6)		646 (18.7)
Medium high risk	17 (16.7)	17 (16.7)		729 (21.1)
Highest risk	60 (58.8)	60 (58.8)		1,998 (57.7)
Hospital			0.989	
Washington Hospital Center	36 (35.3)	37 (36.3)		1,457 (41.6)
Other DC hospitals	63 (61.8)	62 (60.8)		1,930 (55.2)
Maryland hospitals	3 (2.9)	3 (2.9)		105 (3.0)
Virginia hospitals	0 (0.0)	0 (0.0)		7 (0.2)

^a^ *p*-Value from chi-squared and *t*-tests comparing MV intervention and matched control subjects.

^b^ This is the available pool of control subjects for matching, minus the selected control subjects.

MV, Mamatoto Village; SD, standard deviation.

## Results

Propensity score matching demonstrated that participation in the MRHV program was associated with a statistically significant improvement in gestational age (mean increase of 0.34 weeks, *p* < 0.05; see Table 2 and Table 3). Other outcomes favored the program participants but were not statistically significant. Specifically, in the unadjusted regression models, respondents who participated in the MRHV program had a statistically significant (*p* < 0.05) higher mean gestational age compared with controls (b = 0.57, 95% confidence interval [CI]: 0.05–1.10) compared with the matched comparison group. Other unadjusted relationships between participation in the MRHV program and outcomes were in expected directions (e.g., higher birth weight), but these differences compared with participants in the comparison group were not statistically significant.

**Table 2.** Association Between MV Status and Outcomes Through Propensity Score Matching

Outcome	ATET (SE)	*p*-Value
Gestational age	0.34 (0.17)	**0.042** ^a^
Preterm	−0.03 (0.03)	0.300
Birth weight	−22.99 (50.50)	0.649
Low birth weight	0.01 (0.04)	0.815
Level of care	0.00 (0.03)	0.883

Bold values denote statistical significance at the *p* < 0.05 level.

^a^ Statistically significant at 95% CI.

ATET, average treatment effect on the treated; CI, confidence interval; SE, standard error.

**Table 3.** Associations Between MV Status and Outcomes Through Logistic and Linear Regression

	Mean (SD)/proportion (*n*)^a^	*p*-Value	Unadjusted (β/OR)	*p*-Value	Adjusted (β/OR)^b^	*p*-Value
Birth weight^c^		0.717		0.716		0.704
Control	3045.6 (608.8)		—		—	
MV	3073.9 (477.2)		28.25 (−125.0, 181.5)		29.7 (−123.9, 183.2)	
Low birth weight^d^^,^^e^		0.748		0.748		0.753
Control	14.9 (15)		—		—	
MV	13.3 (13)		0.88 (0.39, 1.95)		0.88 (0.39, 1.96)	
Gestational age^c^		**0.011^f^**		**0.011^f^**		**0.016^f^**
Control	38.1 (1.9)		—		—	
MV	38.7 (1.6)		0.64 (0.15, 1.13)		0.60 (0.11, 1.09)	
Preterm^d^		0.142		0.148		0.162
Control	13.8 (13)		—		—	
MV	7.3 (7)		0.49 (0.19, 1.29)		0.50 (0.18, 1.32)	
Level of care^d^		0.654		0.655		0.633
Control	10.9 (11)		—		—	
MV	9.0 (9)		0.81 (0.32, 2.05)		0.79 (0.31, 2.04)	

Bold values denote statistical significance at the *p* < 0.05 level.

^a^ Control, *n* = 98; MV, *n* = 98 for birth weight; Control, *n* = 98; MV, *n* = 98 for gestational age; Control, *n* = 100; MV, *n* = 100 for level of care; SD = standard deviation.

^b^ Adjusted for maternal delivery age, race, delivery hospital category, zip code risk category.

^c^ From linear regression; estimates in β coefficient.

^d^ From logistic regression; estimates in odds ratios (OR).

^e^ No extremely low birth outcomes in MV or control.

^f^ Statistically significant at 95% CI.

OR, odds ratio.

Similarly, in models adjusted for maternal delivery age and race, delivery hospital, and zip code risk category, respondents who participated in the MRHV program had a statistically significant (*p* < 0.05) higher mean gestational age (b = 0.56, 95% CI: 0.03–1.08) compared with the control population. Among other outcomes, MV participants were less likely to deliver a low-birth-weight baby (<2500 g; adjusted odds ratio [aOR] = 0.75, 95% CI: 0.34–1.65) or have a preterm birth (<37 weeks gestation; aOR = 0.70, 95% CI: 0.25–1.94), but these differences were not statistically significant.

## Discussion

The MRHV program is part of a growing movement of perinatal health work that explicitly centers on the lived experiences of Black women from communities that have been disregarded for too long. The founders of MV created a comprehensive training to provide home visitors with new content and skills that build upon their expertise as members of the communities represented in the prioritized population. This study was one of the first to use a rigorous quasi-experimental design to examine the potential effects of their culturally congruent home visiting program. The quantitative results are encouraging: compared with Black women from the same communities, matched on a wide range of variables known to affect maternal and child health outcomes, the MRHV participants had a higher gestational age. Other indicators trended positively but were not statistically significant in this relatively small sample of 100 participants. Still, the outcomes speak to the impact of racism on preterm birth, the need for policy that supports diversification of the perinatal workforce, expansion of access to culturally congruent care, and support for hyperlocal programs that directly address social determinants.

Other communities seeking to address perinatal health inequities have taken similar approaches, underscoring the potential promise that culturally congruent care presents for reducing birth disparities in Black communities. Arteaga et al.^15^ reported on the SisterWeb San Francisco Community Doula Network launched in 2018.^16^ Using a mixed methods design, these authors captured clients’ motivations for seeking culturally congruent community doula support through interviews with 14 clients—all but one identified as Black. Similar elements of the MRHV program can be witnessed in SisterWeb’s community doula model—providing accessible and culturally congruent perinatal support and economic mobility opportunities for the doulas.^17^ Participants described their reasons for seeking doula support, which included a need for a type of care that was in sharp contrast to the medicalized maternity system that they were forced to interact with. Participants also identified specific ways in which having a community doula made their pregnancy, birth, and postpartum experiences different, including providing information about their rights^1^ to respectful and high-quality care, helping with pain management, and building trusting relationships throughout the perinatal period.^15^ Clinicians could benefit from implementing practices demonstrated in community-based programs proven to enhance respectful and culturally congruent care, improve the patient–clinician relationship, and mitigate obstetric violence. Karbeah et al.^18^ pointed to the patient–clinician relationship as one place where racially concordant care might help toward a more equitable and respectful birthing process for Black women.

In the general population, with a doula intervention, there are improvements with a host of maternal and birth outcomes, including preterm delivery.^19^ However, for Black women increased risk for preterm labor is often associated with socioeconomic factors and racism,^20^ causing persistent disparities despite the presence of doula support. In their recent article, Giurgesu et al.^4^ focus on the potential contribution of stress related to neighborhood disadvantage and racial discrimination to preterm delivery. They found that neighborhood-level factors such as vacant housing, poor property conditions, and crime combined with racial discrimination led to increased preterm birth, all of which are factors not typically addressed by doulas trained using rapid training models (i.e., <30 h) established by popular White-led organizations. More attention needs to be given to comprehensive perinatal care models for Black women that are not limited exclusively to doula care. These models and subsequent interventions must address health and social determinants, perinatal mental health, childbirth and parent education, nutrition and food access, and safety.

Recent scholars have called for an intentional shift from focusing on racial differences to centering the voices of Black and Brown people in research through a Reproductive Justice Framework.^4^ The absence of Black voices and lived experiences in setting the research agenda for reproductive health has led to decades of studies that are not asking the right questions nor measuring the right variables.^21^ Examples such as the cross-sector work on the Community Birth Plan Task Force^22^ in Los Angeles County^23^ are important models of how to engage Black women who have experienced preterm birth alongside perinatal health clinicians and community-based organizations serving Black families in cocreating solutions that may reduce disparities in those communities. To date, the literature has not documented the effectiveness of interventions that can shift outcomes at the population level needed to close the maternal and infant mortality gap. Mothers Rising elucidates the need to invest in hyperlocal, community-developed solutions to optimize Black maternal and infant health outcomes and ensure access to respectful, culturally congruent, and high-quality perinatal care.

## Abbreviations Used

MCO: Managed Care Organization
MRHV: Mothers Rising Home Visiting
MV: Mamatoto Village
NICU: neonatal intensive care unit
PCHW: Perinatal Community Health Worker

## Notes

^1^
The National Association to Advance Black Birth created the Black Birth Bill of Rights, an infographic tool, for Black birthing people to confidently exercise their rights to a respectful, equitable, and high-quality maternity care. https://thenaabb.org/black-birthing-bill-of-rights/
